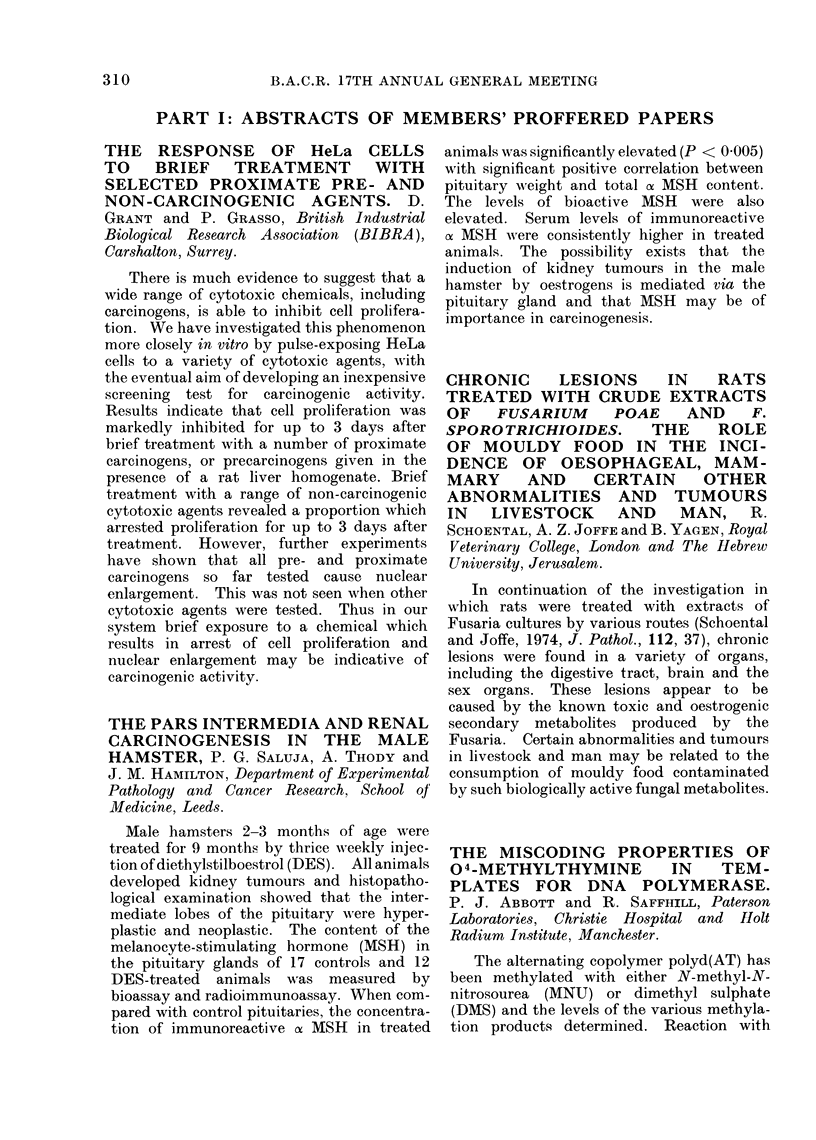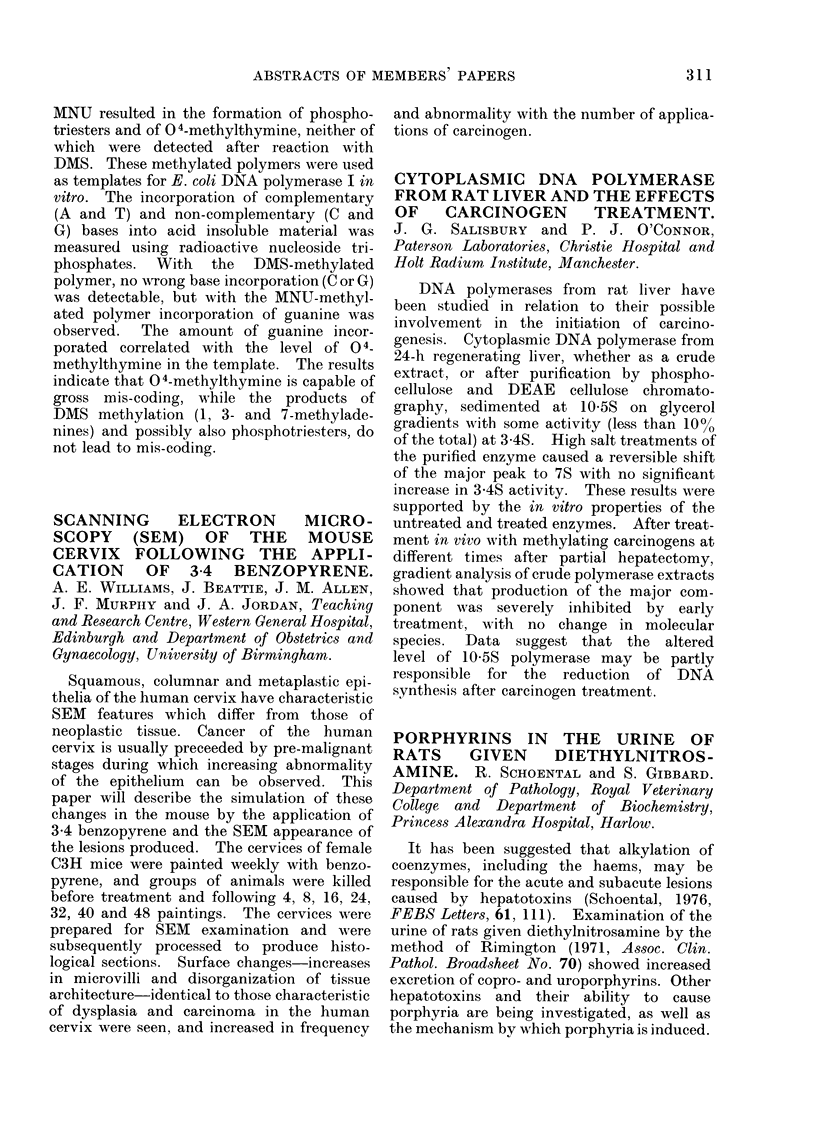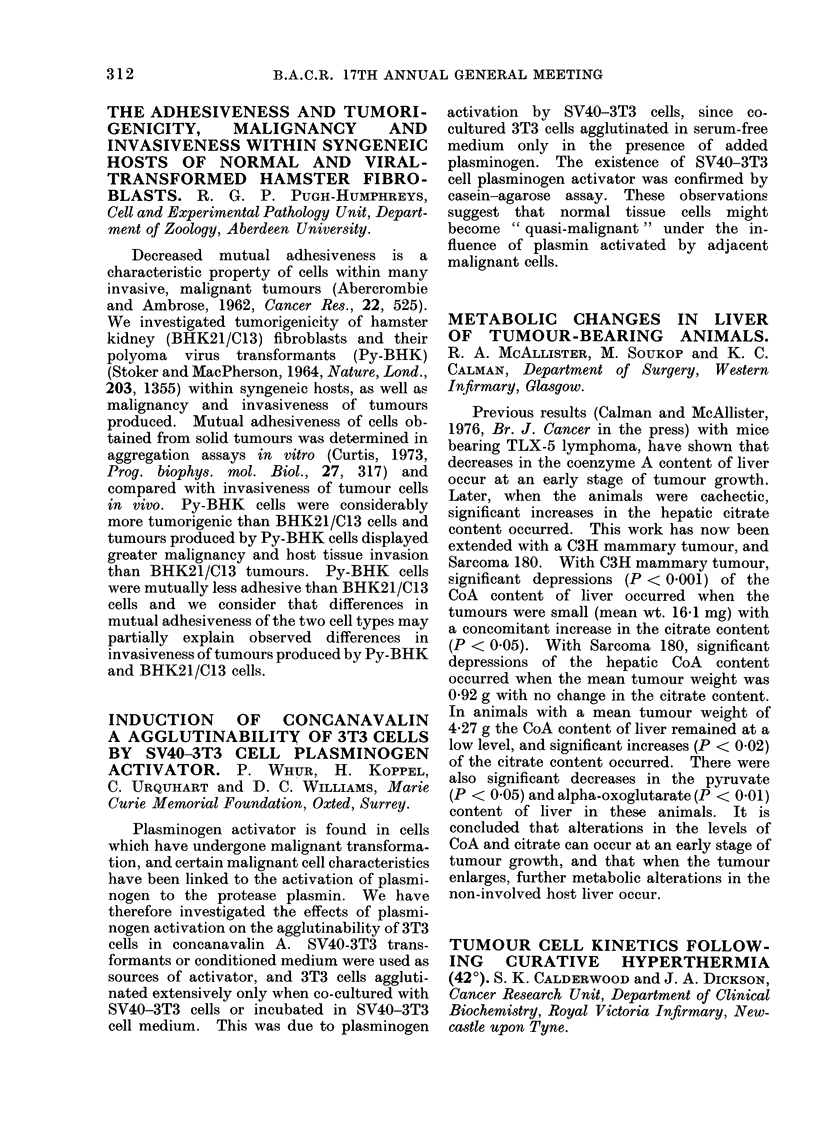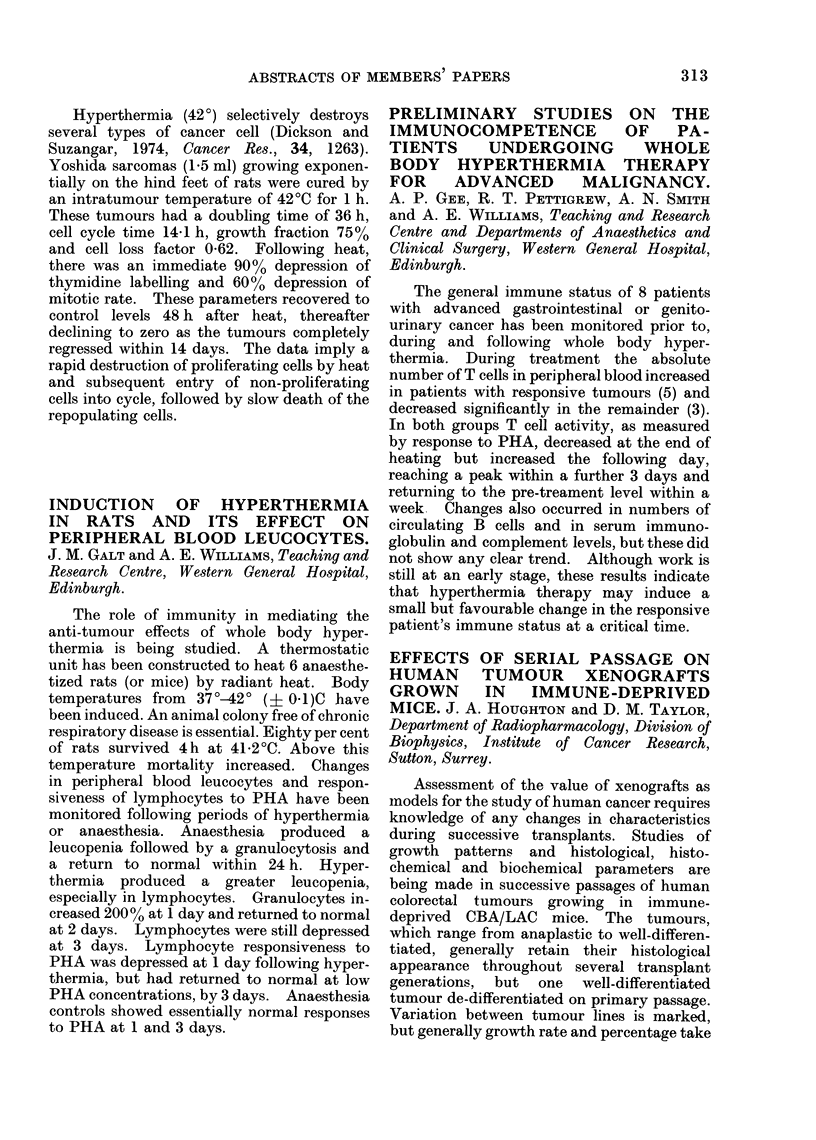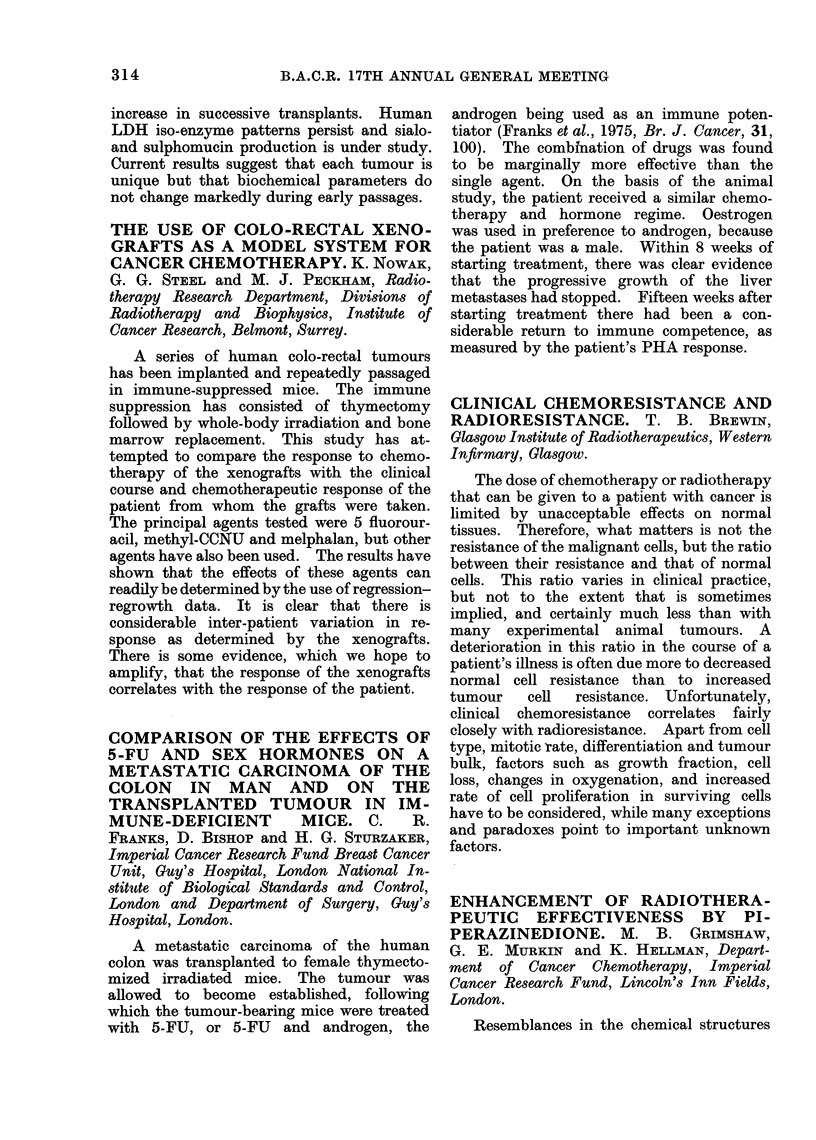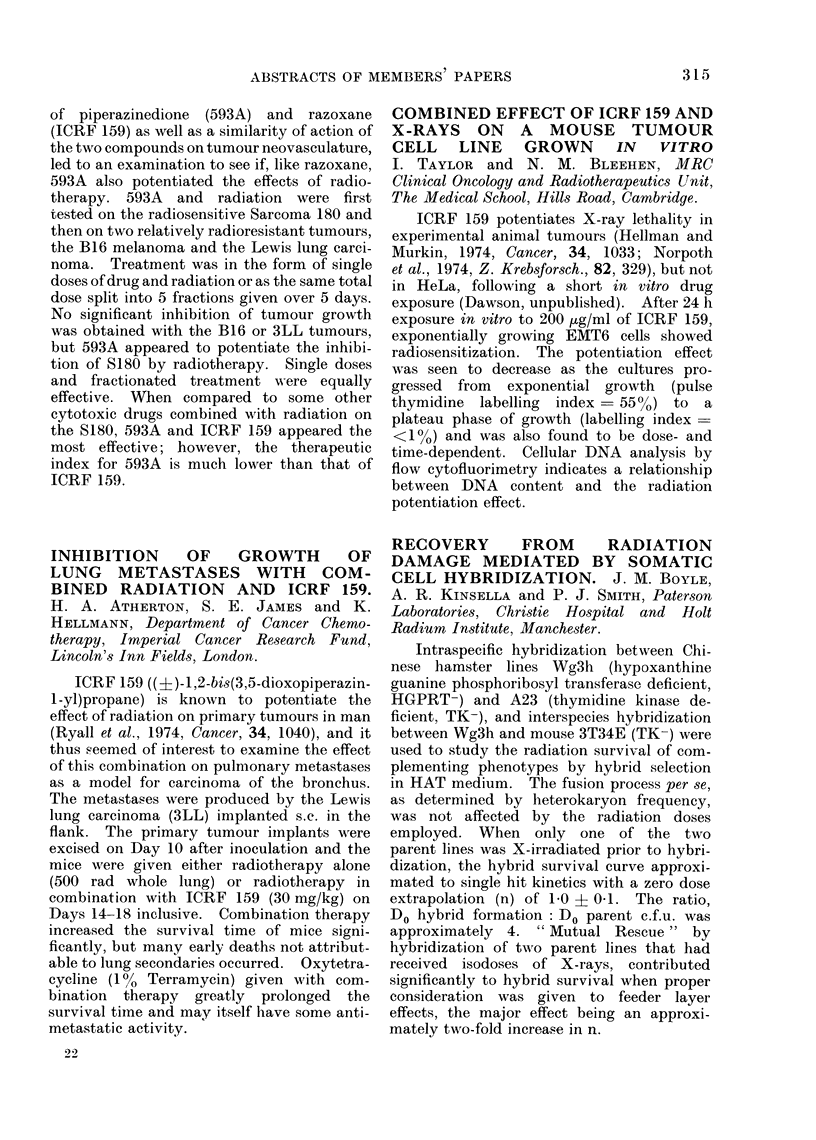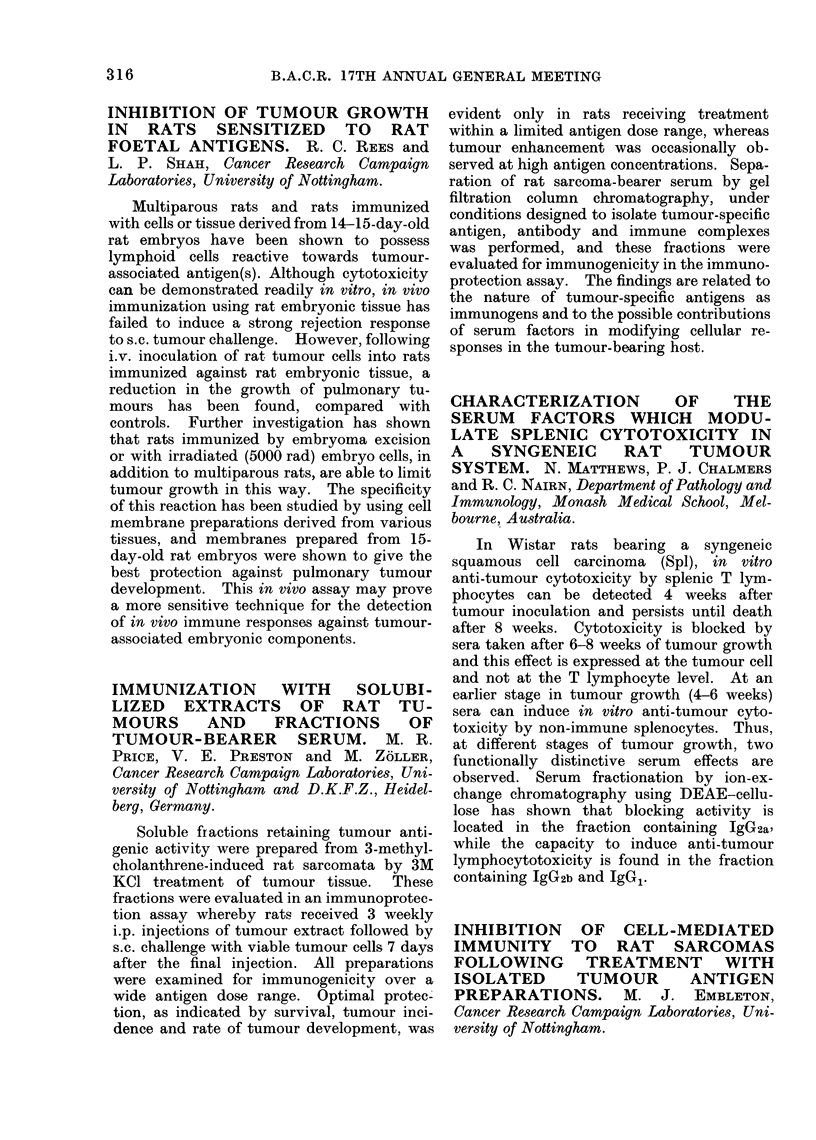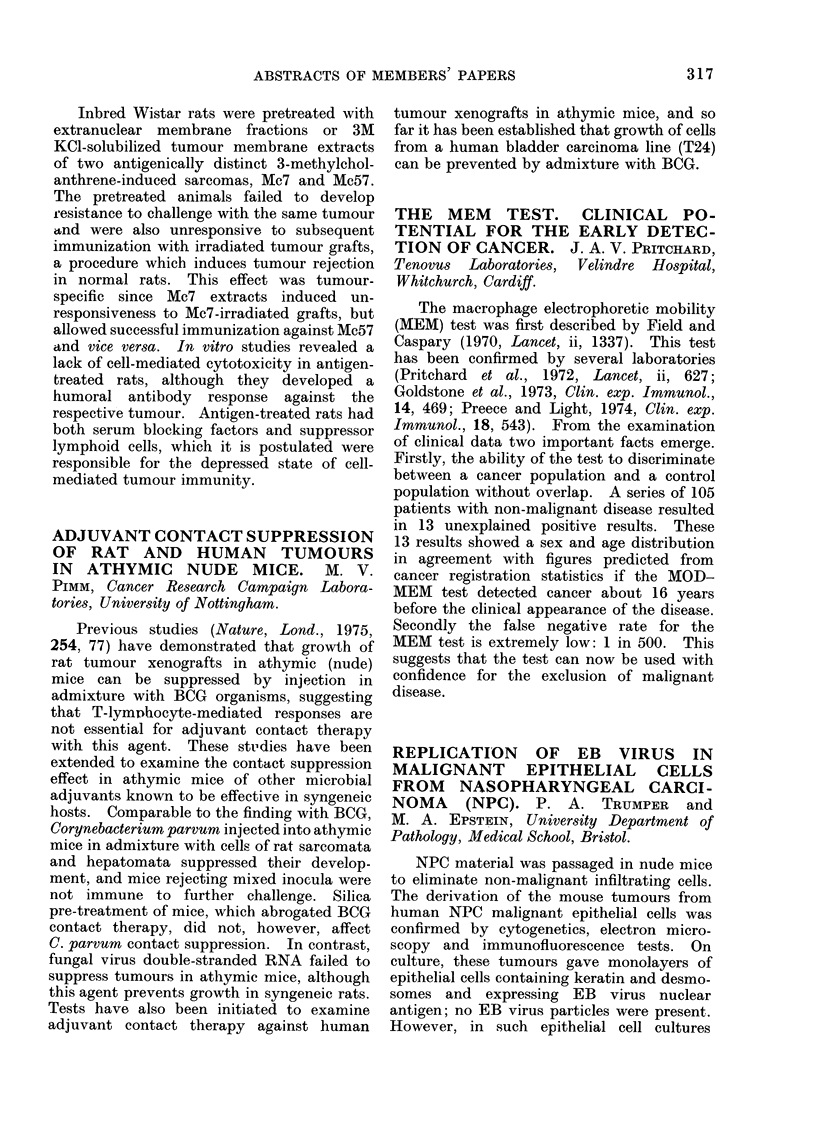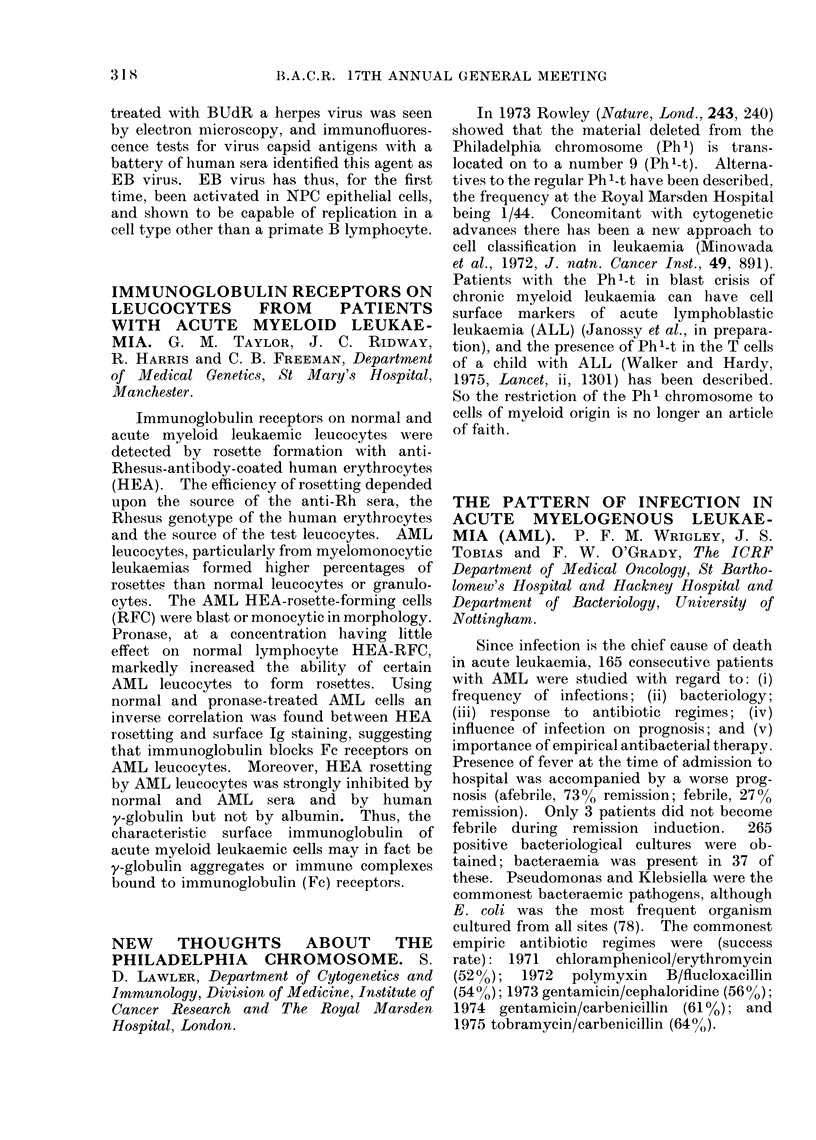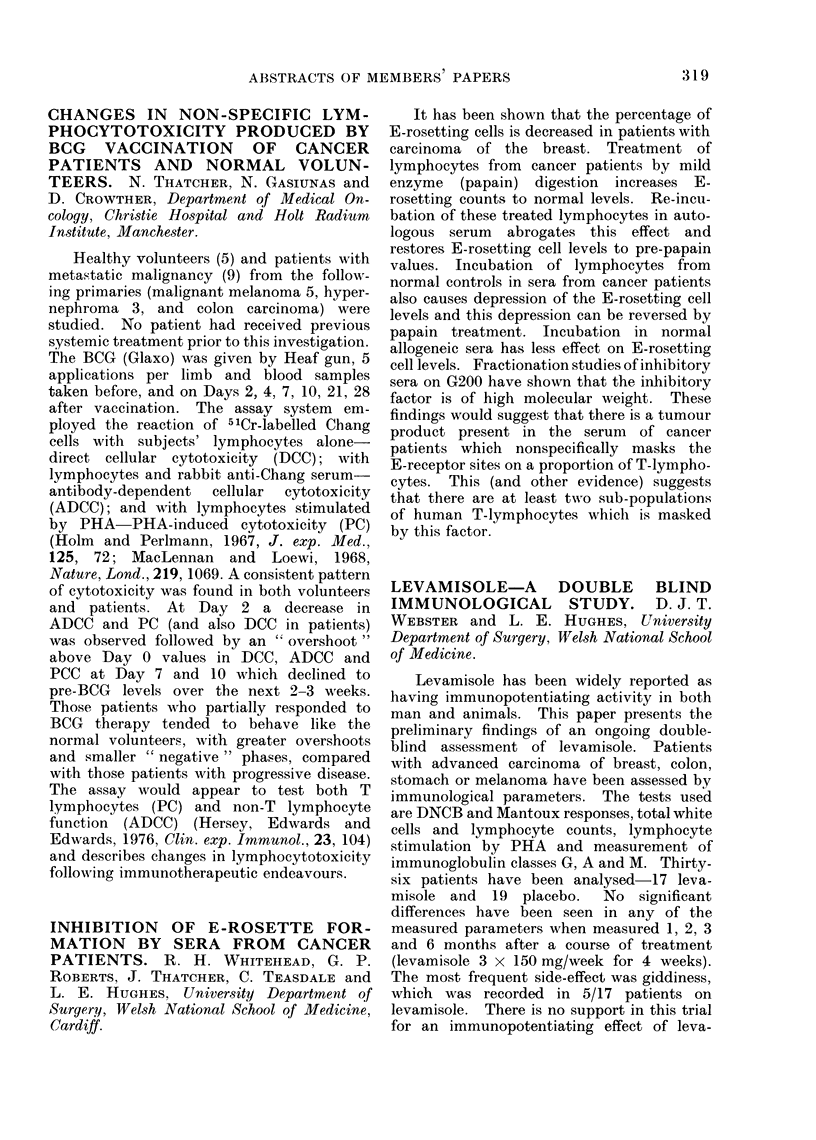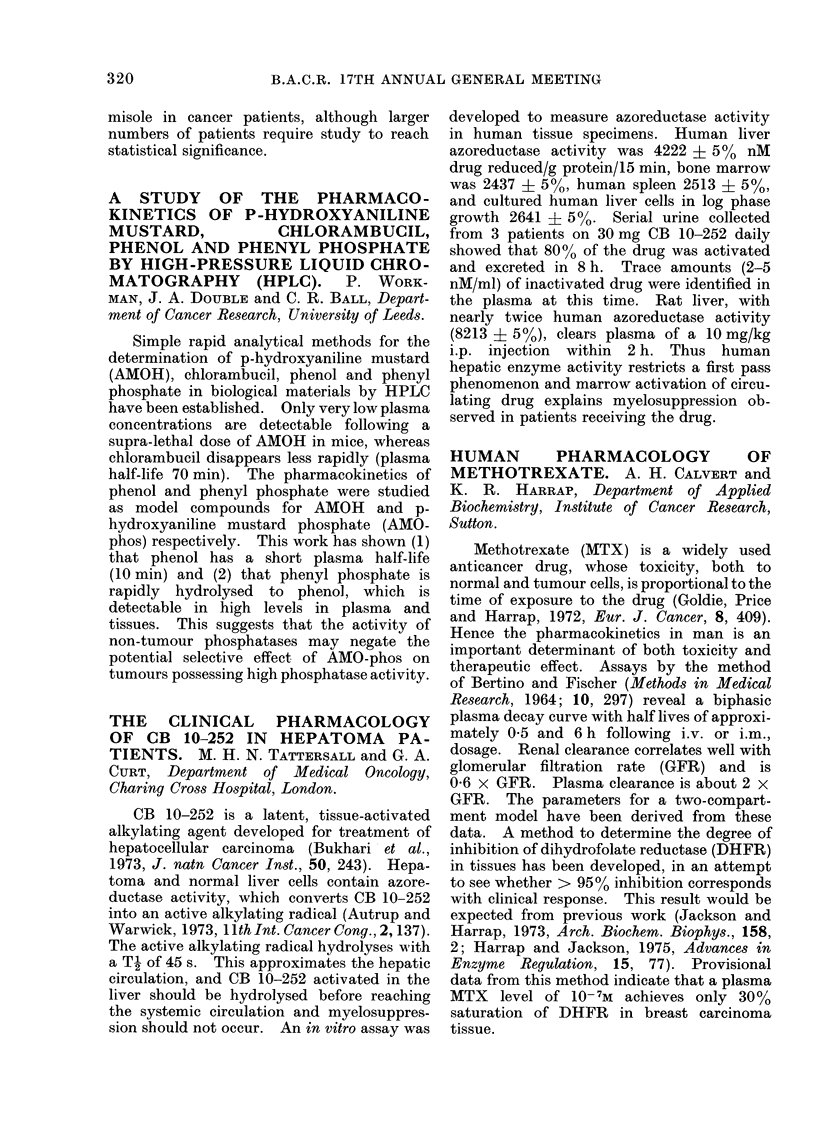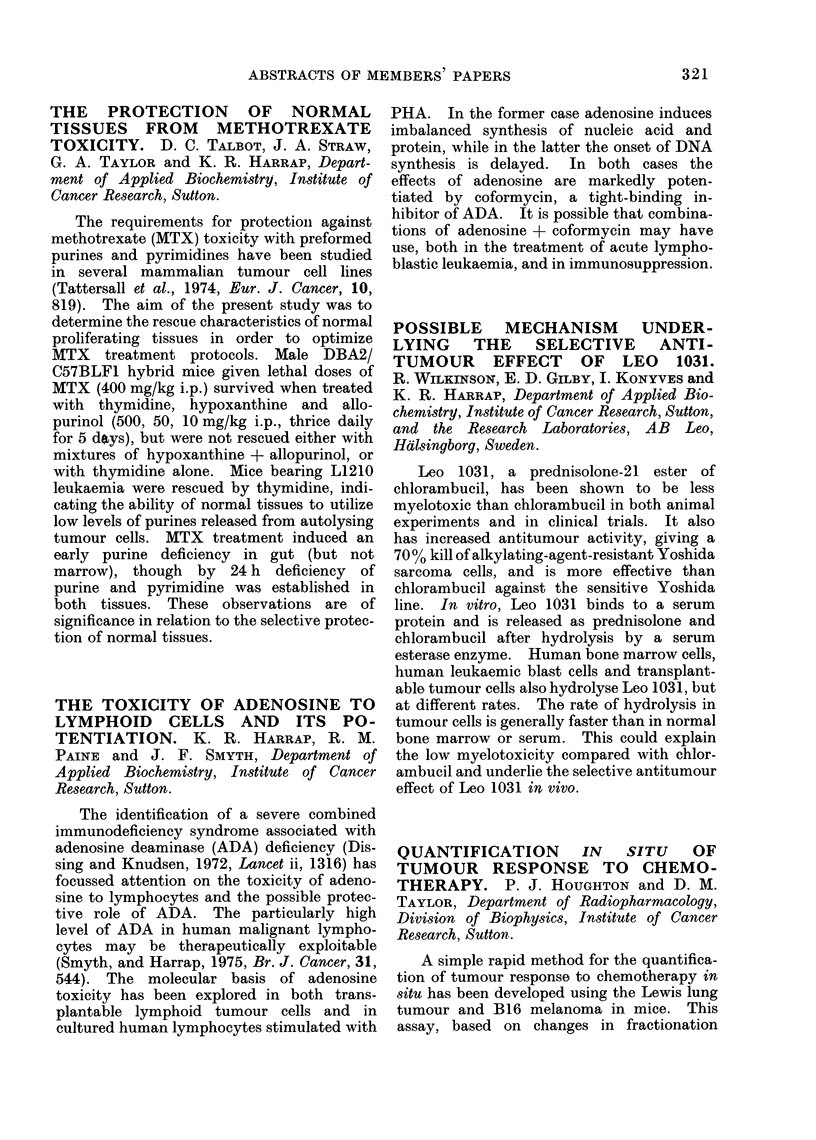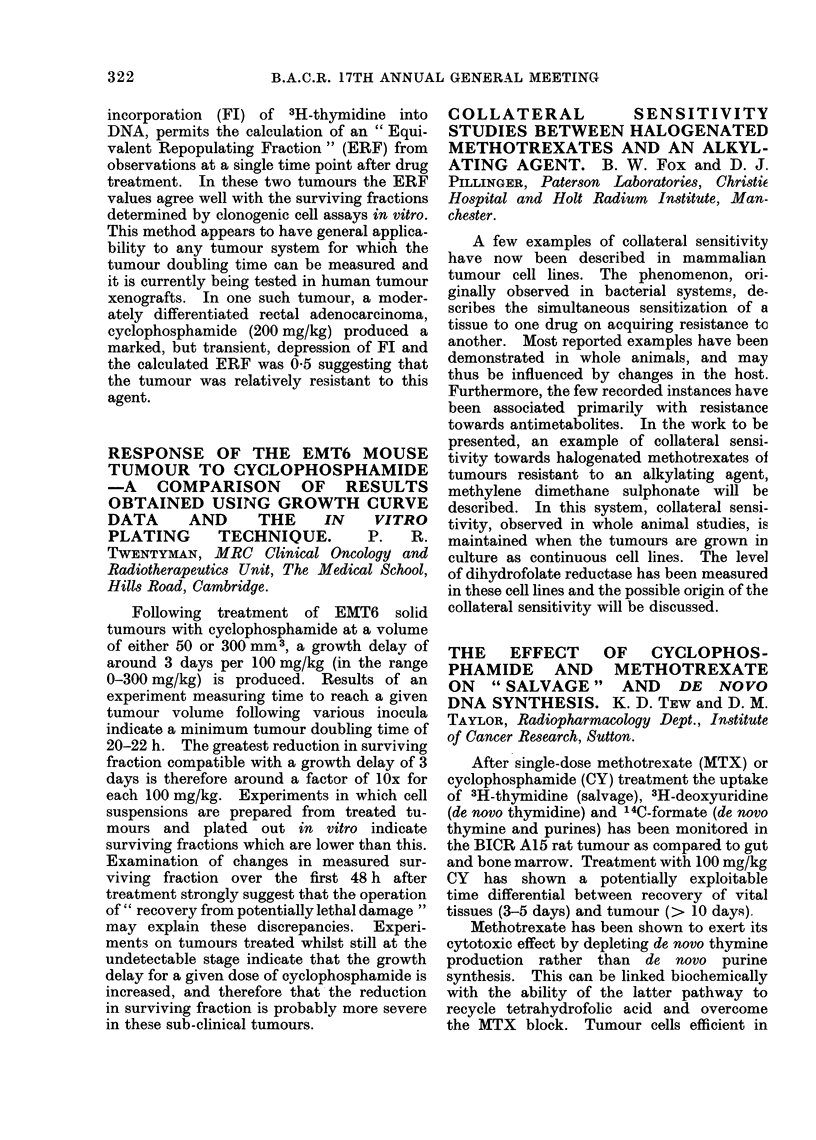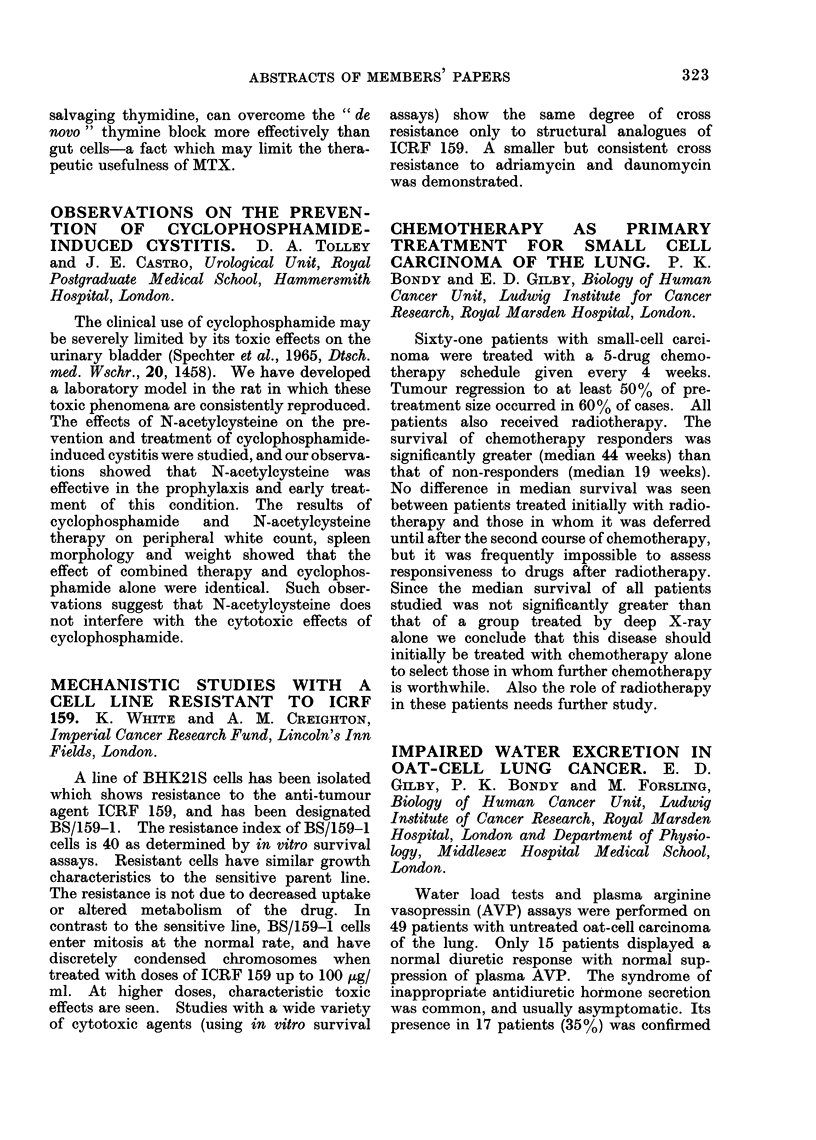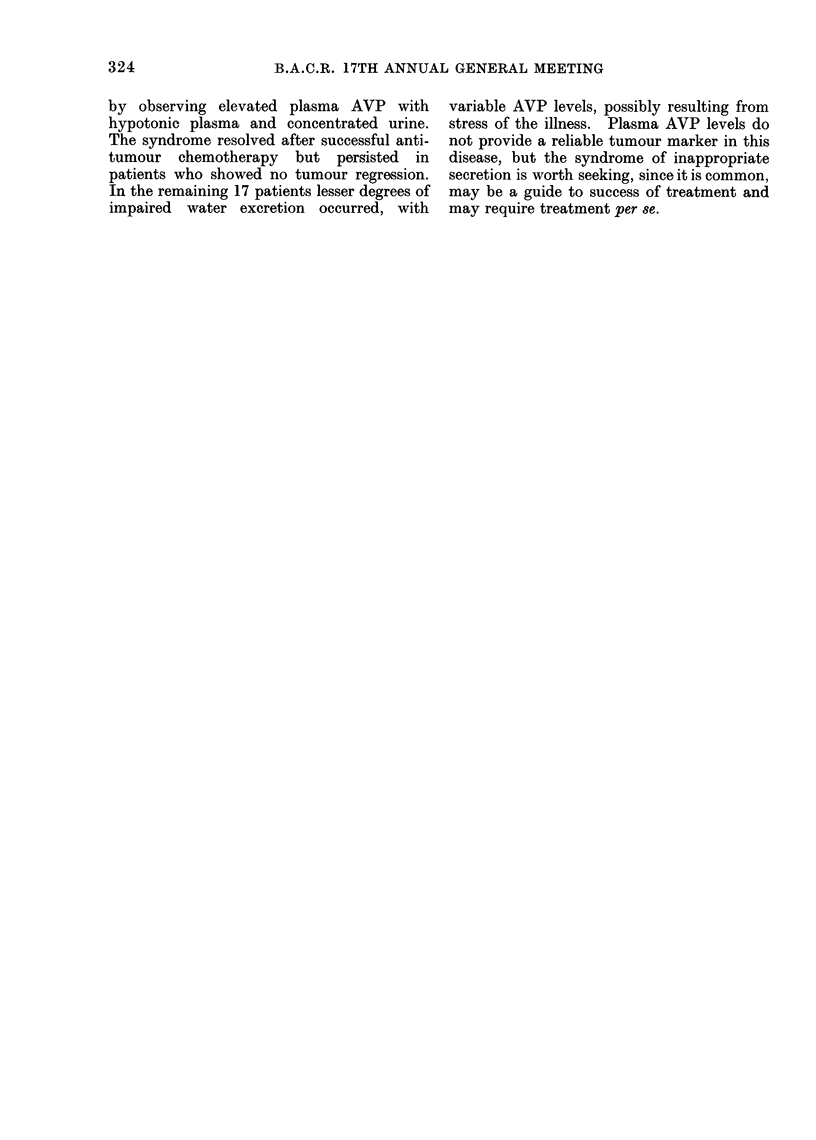# Part I: Abstracts of Members' Proffered Papers

**Published:** 1976-09

**Authors:** 


### PART I: ABSTRACTS OF MEMBERS' PROFFERED PAPERS

### THE RESPONSE OF HeLa CELLS TO BRIEF TREATMENT WITH SELECTED PROXIMATE PRE- AND NON-CARCINOGENIC AGENTS.

D. GRANT and P. GRASSO, *British Industrial Biological Research Association (BIBRA), Carshalton, Surrey.*

There is much evidence to suggest that a wide range of cytotoxic chemicals, including carcinogens, is able to inhibit cell proliferation. We have investigated this phenomenon more closely *in vitro* by pulse-exposing HeLa cells to a variety of cytotoxic agents, with the eventual aim of developing an inexpensive screening test for carcinogenic activity. Results indicate that cell proliferation was markedly inhibited for up to 3 days after brief treatment with a number of proximate carcinogens, or precarcinogens given in the presence of a rat liver homogenate. Brief treatment with a range of non-carcinogenic cytotoxic agents revealed a proportion which arrested proliferation for up to 3 days after treatment. However, further experiments have shown that all pre- and proximate carcinogens so far tested cause nuclear enlargement. This was not seen when other cytotoxic agents were tested. Thus in our system brief exposure to a chemical which results in arrest of cell proliferation and nuclear enlargement may be indicative of carcinogenic activity.

### THE PARS INTERMEDIA AND RENAL CARCINOGENESIS IN THE MALE HAMSTER,

P. G. SALUJA, A. THODY and J. M. HAMILTON, *Department of Experimental Pathology and Cancer Research, School of Medicine, Leeds.*

Male hamsters 2–3 months of age were treated for 9 months by thrice weekly injection of diethylstilboestrol (DES). All animals developed kidney tumours and histopathological examination showed that the intermediate lobes of the pituitary were hyperplastic and neoplastic. The content of the melanocyte-stimulating hormone (MSH) in the pituitary glands of 17 controls and 12 DES-treated animals was measured by bioassay and radioimmunoassay. When compared with control pituitaries, the concentration of immunoreactive $\alpha$ MSH in treated animals was significantly elevated ($P < 0{\cdot}005$) with significant positive correlation between pituitary weight and total $\alpha$ MSH content. The levels of bioactive MSH were also elevated. Serum levels of immunoreactive $\alpha$ MSH were consistently higher in treated animals. The possibility exists that the induction of kidney tumours in the male hamster by oestrogens is mediated *via* the pituitary gland and that MSH may be of importance in carcinogenesis.

### CHRONIC LESIONS IN RATS TREATED WITH CRUDE EXTRACTS OF *FUSARIUM POAE* AND *F. SPOROTRICHIOIDES*. THE ROLE OF MOULDY FOOD IN THE INCIDENCE OF OESOPHAGEAL, MAMMARY AND CERTAIN OTHER ABNORMALITIES AND TUMOURS IN LIVESTOCK AND MAN,

R. SCHOENTAL, A. Z. JOFFE and B. YAGEN, *Royal Veterinary College, London and The Hebrew University, Jerusalem.*

In continuation of the investigation in which rats were treated with extracts of Fusaria cultures by various routes (Schoental and Joffe, 1974, *J. Pathol.*, **112**, 37), chronic lesions were found in a variety of organs, including the digestive tract, brain and the sex organs. These lesions appear to be caused by the known toxic and oestrogenic secondary metabolites produced by the Fusaria. Certain abnormalities and tumours in livestock and man may be related to the consumption of mouldy food contaminated by such biologically active fungal metabolites.

### THE MISCODING PROPERTIES OF O[4]-METHYLTHYMINE IN TEMPLATES FOR DNA POLYMERASE.

P. J. ABBOTT and R. SAFFHILL, *Paterson Laboratories, Christie Hospital and Holt Radium Institute, Manchester.*

The alternating copolymer polyd(AT) has been methylated with either *N*-methyl-*N*-nitrosourea (MNU) or dimethyl sulphate (DMS) and the levels of the various methylation products determined. Reaction with

MNU resulted in the formation of phosphotriesters and of $O^4$-methylthymine, neither of which were detected after reaction with DMS. These methylated polymers were used as templates for *E. coli* DNA polymerase I *in vitro*. The incorporation of complementary (A and T) and non-complementary (C and G) bases into acid insoluble material was measured using radioactive nucleoside triphosphates. With the DMS-methylated polymer, no wrong base incorporation (C or G) was detectable, but with the MNU-methylated polymer incorporation of guanine was observed. The amount of guanine incorporated correlated with the level of $O^4$-methylthymine in the template. The results indicate that $O^4$-methylthymine is capable of gross mis-coding, while the products of DMS methylation (1, 3- and 7-methyladenines) and possibly also phosphotriesters, do not lead to mis-coding.

### SCANNING ELECTRON MICROSCOPY (SEM) OF THE MOUSE CERVIX FOLLOWING THE APPLICATION OF 3·4 BENZOPYRENE.

A. E. Williams, J. Beattie, J. M. Allen, J. F. Murphy and J. A. Jordan, *Teaching and Research Centre, Western General Hospital, Edinburgh and Department of Obstetrics and Gynaecology, University of Birmingham.*

Squamous, columnar and metaplastic epithelia of the human cervix have characteristic SEM features which differ from those of neoplastic tissue. Cancer of the human cervix is usually preceeded by pre-malignant stages during which increasing abnormality of the epithelium can be observed. This paper will describe the simulation of these changes in the mouse by the application of 3·4 benzopyrene and the SEM appearance of the lesions produced. The cervices of female C3H mice were painted weekly with benzopyrene, and groups of animals were killed before treatment and following 4, 8, 16, 24, 32, 40 and 48 paintings. The cervices were prepared for SEM examination and were subsequently processed to produce histological sections. Surface changes—increases in microvilli and disorganization of tissue architecture—identical to those characteristic of dysplasia and carcinoma in the human cervix were seen, and increased in frequency and abnormality with the number of applications of carcinogen.

### CYTOPLASMIC DNA POLYMERASE FROM RAT LIVER AND THE EFFECTS OF CARCINOGEN TREATMENT.

J. G. Salisbury and P. J. O'Connor, *Paterson Laboratories, Christie Hospital and Holt Radium Institute, Manchester.*

DNA polymerases from rat liver have been studied in relation to their possible involvement in the initiation of carcinogenesis. Cytoplasmic DNA polymerase from 24-h regenerating liver, whether as a crude extract, or after purification by phosphocellulose and DEAE cellulose chromatography, sedimented at 10·5S on glycerol gradients with some activity (less than 10% of the total) at 3·4S. High salt treatments of the purified enzyme caused a reversible shift of the major peak to 7S with no significant increase in 3·4S activity. These results were supported by the *in vitro* properties of the untreated and treated enzymes. After treatment *in vivo* with methylating carcinogens at different times after partial hepatectomy, gradient analysis of crude polymerase extracts showed that production of the major component was severely inhibited by early treatment, with no change in molecular species. Data suggest that the altered level of 10·5S polymerase may be partly responsible for the reduction of DNA synthesis after carcinogen treatment.

### PORPHYRINS IN THE URINE OF RATS GIVEN DIETHYLNITROSAMINE.

R. Schoental and S. Gibbard. *Department of Pathology, Royal Veterinary College and Department of Biochemistry, Princess Alexandra Hospital, Harlow.*

It has been suggested that alkylation of coenzymes, including the haems, may be responsible for the acute and subacute lesions caused by hepatotoxins (Schoental, 1976, *FEBS Letters*, **61**, 111). Examination of the urine of rats given diethylnitrosamine by the method of Rimington (1971, *Assoc. Clin. Pathol. Broadsheet No.* **70**) showed increased excretion of copro- and uroporphyrins. Other hepatotoxins and their ability to cause porphyria are being investigated, as well as the mechanism by which porphyria is induced.

**THE ADHESIVENESS AND TUMORIGENICITY, MALIGNANCY AND INVASIVENESS WITHIN SYNGENEIC HOSTS OF NORMAL AND VIRAL-TRANSFORMED HAMSTER FIBROBLASTS.** R. G. P. PUGH-HUMPHREYS, *Cell and Experimental Pathology Unit, Department of Zoology, Aberdeen University.*

Decreased mutual adhesiveness is a characteristic property of cells within many invasive, malignant tumours (Abercrombie and Ambrose, 1962, *Cancer Res.*, **22**, 525). We investigated tumorigenicity of hamster kidney (BHK21/C13) fibroblasts and their polyoma virus transformants (Py-BHK) (Stoker and MacPherson, 1964, *Nature, Lond.*, **203**, 1355) within syngeneic hosts, as well as malignancy and invasiveness of tumours produced. Mutual adhesiveness of cells obtained from solid tumours was determined in aggregation assays *in vitro* (Curtis, 1973, *Prog. biophys. mol. Biol.*, **27**, 317) and compared with invasiveness of tumour cells *in vivo*. Py-BHK cells were considerably more tumorigenic than BHK21/C13 cells and tumours produced by Py-BHK cells displayed greater malignancy and host tissue invasion than BHK21/C13 tumours. Py-BHK cells were mutually less adhesive than BHK21/C13 cells and we consider that differences in mutual adhesiveness of the two cell types may partially explain observed differences in invasiveness of tumours produced by Py-BHK and BHK21/C13 cells.

**INDUCTION OF CONCANAVALIN A AGGLUTINABILITY OF 3T3 CELLS BY SV40–3T3 CELL PLASMINOGEN ACTIVATOR.** P. WHUR, H. KOPPEL, C. URQUHART and D. C. WILLIAMS, *Marie Curie Memorial Foundation, Oxted, Surrey.*

Plasminogen activator is found in cells which have undergone malignant transformation, and certain malignant cell characteristics have been linked to the activation of plasminogen to the protease plasmin. We have therefore investigated the effects of plasminogen activation on the agglutinability of 3T3 cells in concanavalin A. SV40-3T3 transformants or conditioned medium were used as sources of activator, and 3T3 cells agglutinated extensively only when co-cultured with SV40–3T3 cells or incubated in SV40–3T3 cell medium. This was due to plasminogen activation by SV40–3T3 cells, since co-cultured 3T3 cells agglutinated in serum-free medium only in the presence of added plasminogen. The existence of SV40–3T3 cell plasminogen activator was confirmed by casein–agarose assay. These observations suggest that normal tissue cells might become " quasi-malignant " under the influence of plasmin activated by adjacent malignant cells.

**METABOLIC CHANGES IN LIVER OF TUMOUR-BEARING ANIMALS.** R. A. MCALLISTER, M. SOUKOP and K. C. CALMAN, *Department of Surgery, Western Infirmary, Glasgow.*

Previous results (Calman and McAllister, 1976, *Br. J. Cancer* in the press) with mice bearing TLX-5 lymphoma, have shown that decreases in the coenzyme A content of liver occur at an early stage of tumour growth. Later, when the animals were cachectic, significant increases in the hepatic citrate content occurred. This work has now been extended with a C3H mammary tumour, and Sarcoma 180. With C3H mammary tumour, significant depressions ($P < 0{\cdot}001$) of the CoA content of liver occurred when the tumours were small (mean wt. 16·1 mg) with a concomitant increase in the citrate content ($P < 0{\cdot}05$). With Sarcoma 180, significant depressions of the hepatic CoA content occurred when the mean tumour weight was 0·92 g with no change in the citrate content. In animals with a mean tumour weight of 4·27 g the CoA content of liver remained at a low level, and significant increases ($P < 0{\cdot}02$) of the citrate content occurred. There were also significant decreases in the pyruvate ($P < 0{\cdot}05$) and alpha-oxoglutarate ($P < 0{\cdot}01$) content of liver in these animals. It is concluded that alterations in the levels of CoA and citrate can occur at an early stage of tumour growth, and that when the tumour enlarges, further metabolic alterations in the non-involved host liver occur.

**TUMOUR CELL KINETICS FOLLOWING CURATIVE HYPERTHERMIA (42°).** S. K. CALDERWOOD and J. A. DICKSON, *Cancer Research Unit, Department of Clinical Biochemistry, Royal Victoria Infirmary, Newcastle upon Tyne.*

Hyperthermia (42°) selectively destroys several types of cancer cell (Dickson and Suzangar, 1974, *Cancer Res.*, **34**, 1263). Yoshida sarcomas (1·5 ml) growing exponentially on the hind feet of rats were cured by an intratumour temperature of 42°C for 1 h. These tumours had a doubling time of 36 h, cell cycle time 14·1 h, growth fraction 75% and cell loss factor 0·62. Following heat, there was an immediate 90% depression of thymidine labelling and 60% depression of mitotic rate. These parameters recovered to control levels 48 h after heat, thereafter declining to zero as the tumours completely regressed within 14 days. The data imply a rapid destruction of proliferating cells by heat and subsequent entry of non-proliferating cells into cycle, followed by slow death of the repopulating cells.

**INDUCTION OF HYPERTHERMIA IN RATS AND ITS EFFECT ON PERIPHERAL BLOOD LEUCOCYTES.** J. M. Galt and A. E. Williams, *Teaching and Research Centre, Western General Hospital, Edinburgh.*

The role of immunity in mediating the anti-tumour effects of whole body hyperthermia is being studied. A thermostatic unit has been constructed to heat 6 anaesthetized rats (or mice) by radiant heat. Body temperatures from 37°–42° (± 0·1)C have been induced. An animal colony free of chronic respiratory disease is essential. Eighty per cent of rats survived 4 h at 41·2°C. Above this temperature mortality increased. Changes in peripheral blood leucocytes and responsiveness of lymphocytes to PHA have been monitored following periods of hyperthermia or anaesthesia. Anaesthesia produced a leucopenia followed by a granulocytosis and a return to normal within 24 h. Hyperthermia produced a greater leucopenia, especially in lymphocytes. Granulocytes increased 200% at 1 day and returned to normal at 2 days. Lymphocytes were still depressed at 3 days. Lymphocyte responsiveness to PHA was depressed at 1 day following hyperthermia, but had returned to normal at low PHA concentrations, by 3 days. Anaesthesia controls showed essentially normal responses to PHA at 1 and 3 days.

**PRELIMINARY STUDIES ON THE IMMUNOCOMPETENCE OF PATIENTS UNDERGOING WHOLE BODY HYPERTHERMIA THERAPY FOR ADVANCED MALIGNANCY.** A. P. Gee, R. T. Pettigrew, A. N. Smith and A. E. Williams, *Teaching and Research Centre and Departments of Anaesthetics and Clinical Surgery, Western General Hospital, Edinburgh.*

The general immune status of 8 patients with advanced gastrointestinal or genitourinary cancer has been monitored prior to, during and following whole body hyperthermia. During treatment the absolute number of T cells in peripheral blood increased in patients with responsive tumours (5) and decreased significantly in the remainder (3). In both groups T cell activity, as measured by response to PHA, decreased at the end of heating but increased the following day, reaching a peak within a further 3 days and returning to the pre-treament level within a week. Changes also occurred in numbers of circulating B cells and in serum immunoglobulin and complement levels, but these did not show any clear trend. Although work is still at an early stage, these results indicate that hyperthermia therapy may induce a small but favourable change in the responsive patient's immune status at a critical time.

**EFFECTS OF SERIAL PASSAGE ON HUMAN TUMOUR XENOGRAFTS GROWN IN IMMUNE-DEPRIVED MICE.** J. A. Houghton and D. M. Taylor, *Department of Radiopharmacology, Division of Biophysics, Institute of Cancer Research, Sutton, Surrey.*

Assessment of the value of xenografts as models for the study of human cancer requires knowledge of any changes in characteristics during successive transplants. Studies of growth patterns and histological, histochemical and biochemical parameters are being made in successive passages of human colorectal tumours growing in immune-deprived CBA/LAC mice. The tumours, which range from anaplastic to well-differentiated, generally retain their histological appearance throughout several transplant generations, but one well-differentiated tumour de-differentiated on primary passage. Variation between tumour lines is marked, but generally growth rate and percentage take increase in successive transplants. Human LDH iso-enzyme patterns persist and sialo- and sulphomucin production is under study. Current results suggest that each tumour is unique but that biochemical parameters do not change markedly during early passages.

**THE USE OF COLO-RECTAL XENOGRAFTS AS A MODEL SYSTEM FOR CANCER CHEMOTHERAPY.** K. NOWAK, G. G. STEEL and M. J. PECKHAM, *Radiotherapy Research Department, Divisions of Radiotherapy and Biophysics, Institute of Cancer Research, Belmont, Surrey.*

A series of human colo-rectal tumours has been implanted and repeatedly passaged in immune-suppressed mice. The immune suppression has consisted of thymectomy followed by whole-body irradiation and bone marrow replacement. This study has attempted to compare the response to chemotherapy of the xenografts with the clinical course and chemotherapeutic response of the patient from whom the grafts were taken. The principal agents tested were 5 fluorouracil, methyl-CCNU and melphalan, but other agents have also been used. The results have shown that the effects of these agents can readily be determined by the use of regression–regrowth data. It is clear that there is considerable inter-patient variation in response as determined by the xenografts. There is some evidence, which we hope to amplify, that the response of the xenografts correlates with the response of the patient.

**COMPARISON OF THE EFFECTS OF 5-FU AND SEX HORMONES ON A METASTATIC CARCINOMA OF THE COLON IN MAN AND ON THE TRANSPLANTED TUMOUR IN IMMUNE-DEFICIENT MICE.** C. R. FRANKS, D. BISHOP and H. G. STURZAKER, *Imperial Cancer Research Fund Breast Cancer Unit, Guy's Hospital, London National Institute of Biological Standards and Control, London and Department of Surgery, Guy's Hospital, London.*

A metastatic carcinoma of the human colon was transplanted to female thymectomized irradiated mice. The tumour was allowed to become established, following which the tumour-bearing mice were treated with 5-FU, or 5-FU and androgen, the androgen being used as an immune potentiator (Franks *et al.*, 1975, *Br. J. Cancer*, **31**, 100). The combination of drugs was found to be marginally more effective than the single agent. On the basis of the animal study, the patient received a similar chemotherapy and hormone regime. Oestrogen was used in preference to androgen, because the patient was a male. Within 8 weeks of starting treatment, there was clear evidence that the progressive growth of the liver metastases had stopped. Fifteen weeks after starting treatment there had been a considerable return to immune competence, as measured by the patient's PHA response.

**CLINICAL CHEMORESISTANCE AND RADIORESISTANCE.** T. B. BREWIN, *Glasgow Institute of Radiotherapeutics, Western Infirmary, Glasgow.*

The dose of chemotherapy or radiotherapy that can be given to a patient with cancer is limited by unacceptable effects on normal tissues. Therefore, what matters is not the resistance of the malignant cells, but the ratio between their resistance and that of normal cells. This ratio varies in clinical practice, but not to the extent that is sometimes implied, and certainly much less than with many experimental animal tumours. A deterioration in this ratio in the course of a patient's illness is often due more to decreased normal cell resistance than to increased tumour cell resistance. Unfortunately, clinical chemoresistance correlates fairly closely with radioresistance. Apart from cell type, mitotic rate, differentiation and tumour bulk, factors such as growth fraction, cell loss, changes in oxygenation, and increased rate of cell proliferation in surviving cells have to be considered, while many exceptions and paradoxes point to important unknown factors.

**ENHANCEMENT OF RADIOTHERAPEUTIC EFFECTIVENESS BY PIPERAZINEDIONE.** M. B. GRIMSHAW, G. E. MURKIN and K. HELLMAN, *Department of Cancer Chemotherapy, Imperial Cancer Research Fund, Lincoln's Inn Fields, London.*

Resemblances in the chemical structures of piperazinedione (593A) and razoxane (ICRF 159) as well as a similarity of action of the two compounds on tumour neovasculature, led to an examination to see if, like razoxane, 593A also potentiated the effects of radiotherapy. 593A and radiation were first tested on the radiosensitive Sarcoma 180 and then on two relatively radioresistant tumours, the B16 melanoma and the Lewis lung carcinoma. Treatment was in the form of single doses of drug and radiation or as the same total dose split into 5 fractions given over 5 days. No significant inhibition of tumour growth was obtained with the B16 or 3LL tumours, but 593A appeared to potentiate the inhibition of S180 by radiotherapy. Single doses and fractionated treatment were equally effective. When compared to some other cytotoxic drugs combined with radiation on the S180, 593A and ICRF 159 appeared the most effective; however, the therapeutic index for 593A is much lower than that of ICRF 159.

**INHIBITION OF GROWTH OF LUNG METASTASES WITH COMBINED RADIATION AND ICRF 159.** H. A. ATHERTON, S. E. JAMES and K. HELLMANN, *Department of Cancer Chemotherapy, Imperial Cancer Research Fund, Lincoln's Inn Fields, London.*

ICRF 159 ((±)-1,2-*bis*(3,5-dioxopiperazin-1-yl)propane) is known to potentiate the effect of radiation on primary tumours in man (Ryall *et al.*, 1974, *Cancer*, **34**, 1040), and it thus seemed of interest to examine the effect of this combination on pulmonary metastases as a model for carcinoma of the bronchus. The metastases were produced by the Lewis lung carcinoma (3LL) implanted s.c. in the flank. The primary tumour implants were excised on Day 10 after inoculation and the mice were given either radiotherapy alone (500 rad whole lung) or radiotherapy in combination with ICRF 159 (30 mg/kg) on Days 14–18 inclusive. Combination therapy increased the survival time of mice significantly, but many early deaths not attributable to lung secondaries occurred. Oxytetracycline (1% Terramycin) given with combination therapy greatly prolonged the survival time and may itself have some anti-metastatic activity.

**COMBINED EFFECT OF ICRF 159 AND X-RAYS ON A MOUSE TUMOUR CELL LINE GROWN *IN VITRO*** I. TAYLOR and N. M. BLEEHEN, *MRC Clinical Oncology and Radiotherapeutics Unit, The Medical School, Hills Road, Cambridge.*

ICRF 159 potentiates X-ray lethality in experimental animal tumours (Hellman and Murkin, 1974, *Cancer*, **34**, 1033; Norpoth *et al.*, 1974, *Z. Krebsforsch.*, **82**, 329), but not in HeLa, following a short *in vitro* drug exposure (Dawson, unpublished). After 24 h exposure *in vitro* to 200 μg/ml of ICRF 159, exponentially growing EMT6 cells showed radiosensitization. The potentiation effect was seen to decrease as the cultures progressed from exponential growth (pulse thymidine labelling index = 55%) to a plateau phase of growth (labelling index = <1%) and was also found to be dose- and time-dependent. Cellular DNA analysis by flow cytofluorimetry indicates a relationship between DNA content and the radiation potentiation effect.

**RECOVERY FROM RADIATION DAMAGE MEDIATED BY SOMATIC CELL HYBRIDIZATION.** J. M. BOYLE, A. R. KINSELLA and P. J. SMITH, *Paterson Laboratories, Christie Hospital and Holt Radium Institute, Manchester.*

Intraspecific hybridization between Chinese hamster lines Wg3h (hypoxanthine guanine phosphoribosyl transferase deficient, $HGPRT^-$) and A23 (thymidine kinase deficient, $TK^-$), and interspecies hybridization between Wg3h and mouse 3T34E ($TK^-$) were used to study the radiation survival of complementing phenotypes by hybrid selection in HAT medium. The fusion process *per se*, as determined by heterokaryon frequency, was not affected by the radiation doses employed. When only one of the two parent lines was X-irradiated prior to hybridization, the hybrid survival curve approximated to single hit kinetics with a zero dose extrapolation (n) of $1 \cdot 0 \pm 0 \cdot 1$. The ratio, $D_0$ hybrid formation : $D_0$ parent c.f.u. was approximately 4. "Mutual Rescue" by hybridization of two parent lines that had received isodoses of X-rays, contributed significantly to hybrid survival when proper consideration was given to feeder layer effects, the major effect being an approximately two-fold increase in n.

**INHIBITION OF TUMOUR GROWTH IN RATS SENSITIZED TO RAT FOETAL ANTIGENS.** R. C. REES and L. P. SHAH, *Cancer Research Campaign Laboratories, University of Nottingham.*

Multiparous rats and rats immunized with cells or tissue derived from 14–15-day-old rat embryos have been shown to possess lymphoid cells reactive towards tumour-associated antigen(s). Although cytotoxicity can be demonstrated readily *in vitro*, *in vivo* immunization using rat embryonic tissue has failed to induce a strong rejection response to s.c. tumour challenge. However, following i.v. inoculation of rat tumour cells into rats immunized against rat embryonic tissue, a reduction in the growth of pulmonary tumours has been found, compared with controls. Further investigation has shown that rats immunized by embryoma excision or with irradiated (5000 rad) embryo cells, in addition to multiparous rats, are able to limit tumour growth in this way. The specificity of this reaction has been studied by using cell membrane preparations derived from various tissues, and membranes prepared from 15-day-old rat embryos were shown to give the best protection against pulmonary tumour development. This *in vivo* assay may prove a more sensitive technique for the detection of *in vivo* immune responses against tumour-associated embryonic components.

**IMMUNIZATION WITH SOLUBILIZED EXTRACTS OF RAT TUMOURS AND FRACTIONS OF TUMOUR-BEARER SERUM.** M. R. PRICE, V. E. PRESTON and M. ZÖLLER, *Cancer Research Campaign Laboratories, University of Nottingham and D.K.F.Z., Heidelberg, Germany.*

Soluble fractions retaining tumour antigenic activity were prepared from 3-methylcholanthrene-induced rat sarcomata by 3M KCl treatment of tumour tissue. These fractions were evaluated in an immunoprotection assay whereby rats received 3 weekly i.p. injections of tumour extract followed by s.c. challenge with viable tumour cells 7 days after the final injection. All preparations were examined for immunogenicity over a wide antigen dose range. Optimal protection, as indicated by survival, tumour incidence and rate of tumour development, was evident only in rats receiving treatment within a limited antigen dose range, whereas tumour enhancement was occasionally observed at high antigen concentrations. Separation of rat sarcoma-bearer serum by gel filtration column chromatography, under conditions designed to isolate tumour-specific antigen, antibody and immune complexes was performed, and these fractions were evaluated for immunogenicity in the immunoprotection assay. The findings are related to the nature of tumour-specific antigens as immunogens and to the possible contributions of serum factors in modifying cellular responses in the tumour-bearing host.

**CHARACTERIZATION OF THE SERUM FACTORS WHICH MODULATE SPLENIC CYTOTOXICITY IN A SYNGENEIC RAT TUMOUR SYSTEM.** N. MATTHEWS, P. J. CHALMERS and R. C. NAIRN, *Department of Pathology and Immunology, Monash Medical School, Melbourne, Australia.*

In Wistar rats bearing a syngeneic squamous cell carcinoma (Spl), *in vitro* anti-tumour cytotoxicity by splenic T lymphocytes can be detected 4 weeks after tumour inoculation and persists until death after 8 weeks. Cytotoxicity is blocked by sera taken after 6–8 weeks of tumour growth and this effect is expressed at the tumour cell and not at the T lymphocyte level. At an earlier stage in tumour growth (4–6 weeks) sera can induce *in vitro* anti-tumour cytotoxicity by non-immune splenocytes. Thus, at different stages of tumour growth, two functionally distinctive serum effects are observed. Serum fractionation by ion-exchange chromatography using DEAE–cellulose has shown that blocking activity is located in the fraction containing $IgG_{2a}$, while the capacity to induce anti-tumour lymphocytotoxicity is found in the fraction containing $IgG_{2b}$ and $IgG_1$.

**INHIBITION OF CELL-MEDIATED IMMUNITY TO RAT SARCOMAS FOLLOWING TREATMENT WITH ISOLATED TUMOUR ANTIGEN PREPARATIONS.** M. J. EMBLETON, *Cancer Research Campaign Laboratories, University of Nottingham.*

Inbred Wistar rats were pretreated with extranuclear membrane fractions or 3M KCl-solubilized tumour membrane extracts of two antigenically distinct 3-methylcholanthrene-induced sarcomas, Mc7 and Mc57. The pretreated animals failed to develop resistance to challenge with the same tumour and were also unresponsive to subsequent immunization with irradiated tumour grafts, a procedure which induces tumour rejection in normal rats. This effect was tumour-specific since Mc7 extracts induced unresponsiveness to Mc7-irradiated grafts, but allowed successful immunization against Mc57 and *vice versa*. *In vitro* studies revealed a lack of cell-mediated cytotoxicity in antigen-treated rats, although they developed a humoral antibody response against the respective tumour. Antigen-treated rats had both serum blocking factors and suppressor lymphoid cells, which it is postulated were responsible for the depressed state of cell-mediated tumour immunity.

**ADJUVANT CONTACT SUPPRESSION OF RAT AND HUMAN TUMOURS IN ATHYMIC NUDE MICE.** M. V. PIMM, *Cancer Research Campaign Laboratories, University of Nottingham.*

Previous studies (*Nature, Lond.*, 1975, **254**, 77) have demonstrated that growth of rat tumour xenografts in athymic (nude) mice can be suppressed by injection in admixture with BCG organisms, suggesting that T-lymphocyte-mediated responses are not essential for adjuvant contact therapy with this agent. These studies have been extended to examine the contact suppression effect in athymic mice of other microbial adjuvants known to be effective in syngeneic hosts. Comparable to the finding with BCG, *Corynebacterium parvum* injected into athymic mice in admixture with cells of rat sarcomata and hepatomata suppressed their development, and mice rejecting mixed inocula were not immune to further challenge. Silica pre-treatment of mice, which abrogated BCG contact therapy, did not, however, affect *C. parvum* contact suppression. In contrast, fungal virus double-stranded RNA failed to suppress tumours in athymic mice, although this agent prevents growth in syngeneic rats. Tests have also been initiated to examine adjuvant contact therapy against human tumour xenografts in athymic mice, and so far it has been established that growth of cells from a human bladder carcinoma line (T24) can be prevented by admixture with BCG.

**THE MEM TEST. CLINICAL POTENTIAL FOR THE EARLY DETECTION OF CANCER.** J. A. V. PRITCHARD, *Tenovus Laboratories, Velindre Hospital, Whitchurch, Cardiff.*

The macrophage electrophoretic mobility (MEM) test was first described by Field and Caspary (1970, *Lancet*, ii, 1337). This test has been confirmed by several laboratories (Pritchard *et al.*, 1972, *Lancet*, ii, 627; Goldstone *et al.*, 1973, *Clin. exp. Immunol.*, **14**, 469; Preece and Light, 1974, *Clin. exp. Immunol.*, **18**, 543). From the examination of clinical data two important facts emerge. Firstly, the ability of the test to discriminate between a cancer population and a control population without overlap. A series of 105 patients with non-malignant disease resulted in 13 unexplained positive results. These 13 results showed a sex and age distribution in agreement with figures predicted from cancer registration statistics if the MOD–MEM test detected cancer about 16 years before the clinical appearance of the disease. Secondly the false negative rate for the MEM test is extremely low: 1 in 500. This suggests that the test can now be used with confidence for the exclusion of malignant disease.

**REPLICATION OF EB VIRUS IN MALIGNANT EPITHELIAL CELLS FROM NASOPHARYNGEAL CARCINOMA (NPC).** P. A. TRUMPER and M. A. EPSTEIN, *University Department of Pathology, Medical School, Bristol.*

NPC material was passaged in nude mice to eliminate non-malignant infiltrating cells. The derivation of the mouse tumours from human NPC malignant epithelial cells was confirmed by cytogenetics, electron microscopy and immunofluorescence tests. On culture, these tumours gave monolayers of epithelial cells containing keratin and desmosomes and expressing EB virus nuclear antigen; no EB virus particles were present. However, in such epithelial cell cultures treated with BUdR a herpes virus was seen by electron microscopy, and immunofluorescence tests for virus capsid antigens with a battery of human sera identified this agent as EB virus. EB virus has thus, for the first time, been activated in NPC epithelial cells, and shown to be capable of replication in a cell type other than a primate B lymphocyte.

**IMMUNOGLOBULIN RECEPTORS ON LEUCOCYTES FROM PATIENTS WITH ACUTE MYELOID LEUKAEMIA.** G. M. TAYLOR, J. C. RIDWAY, R. HARRIS and C. B. FREEMAN, *Department of Medical Genetics, St Mary's Hospital, Manchester.*

Immunoglobulin receptors on normal and acute myeloid leukaemic leucocytes were detected by rosette formation with anti-Rhesus-antibody-coated human erythrocytes (HEA). The efficiency of rosetting depended upon the source of the anti-Rh sera, the Rhesus genotype of the human erythrocytes and the source of the test leucocytes. AML leucocytes, particularly from myelomonocytic leukaemias formed higher percentages of rosettes than normal leucocytes or granulocytes. The AML HEA-rosette-forming cells (RFC) were blast or monocytic in morphology. Pronase, at a concentration having little effect on normal lymphocyte HEA-RFC, markedly increased the ability of certain AML leucocytes to form rosettes. Using normal and pronase-treated AML cells an inverse correlation was found between HEA rosetting and surface Ig staining, suggesting that immunoglobulin blocks Fc receptors on AML leucocytes. Moreover, HEA rosetting by AML leucocytes was strongly inhibited by normal and AML sera and by human $\gamma$-globulin but not by albumin. Thus, the characteristic surface immunoglobulin of acute myeloid leukaemic cells may in fact be $\gamma$-globulin aggregates or immune complexes bound to immunoglobulin (Fc) receptors.

**NEW THOUGHTS ABOUT THE PHILADELPHIA CHROMOSOME.** S. D. LAWLER, *Department of Cytogenetics and Immunology, Division of Medicine, Institute of Cancer Research and The Royal Marsden Hospital, London.*

In 1973 Rowley (*Nature, Lond.*, **243**, 240) showed that the material deleted from the Philadelphia chromosome ($Ph^1$) is translocated on to a number 9 ($Ph^1$-t). Alternatives to the regular $Ph^1$-t have been described, the frequency at the Royal Marsden Hospital being 1/44. Concomitant with cytogenetic advances there has been a new approach to cell classification in leukaemia (Minowada *et al.*, 1972, *J. natn. Cancer Inst.*, **49**, 891). Patients with the $Ph^1$-t in blast crisis of chronic myeloid leukaemia can have cell surface markers of acute lymphoblastic leukaemia (ALL) (Janossy *et al.*, in preparation), and the presence of $Ph^1$-t in the T cells of a child with ALL (Walker and Hardy, 1975, *Lancet*, ii, 1301) has been described. So the restriction of the $Ph^1$ chromosome to cells of myeloid origin is no longer an article of faith.

**THE PATTERN OF INFECTION IN ACUTE MYELOGENOUS LEUKAEMIA (AML).** P. F. M. WRIGLEY, J. S. TOBIAS and F. W. O'GRADY, *The ICRF Department of Medical Oncology, St Bartholomew's Hospital and Hackney Hospital and Department of Bacteriology, University of Nottingham.*

Since infection is the chief cause of death in acute leukaemia, 165 consecutive patients with AML were studied with regard to: (i) frequency of infections; (ii) bacteriology; (iii) response to antibiotic regimes; (iv) influence of infection on prognosis; and (v) importance of empirical antibacterial therapy. Presence of fever at the time of admission to hospital was accompanied by a worse prognosis (afebrile, 73% remission; febrile, 27% remission). Only 3 patients did not become febrile during remission induction. 265 positive bacteriological cultures were obtained; bacteraemia was present in 37 of these. Pseudomonas and Klebsiella were the commonest bacteraemic pathogens, although *E. coli* was the most frequent organism cultured from all sites (78). The commonest empiric antibiotic regimes were (success rate): 1971 chloramphenicol/erythromycin (52%); 1972 polymyxin B/flucloxacillin (54%); 1973 gentamicin/cephaloridine (56%); 1974 gentamicin/carbenicillin (61%); and 1975 tobramycin/carbenicillin (64%).

**CHANGES IN NON-SPECIFIC LYMPHOCYTOTOXICITY PRODUCED BY BCG VACCINATION OF CANCER PATIENTS AND NORMAL VOLUNTEERS.** N. THATCHER, N. GASIUNAS and D. CROWTHER, *Department of Medical Oncology, Christie Hospital and Holt Radium Institute, Manchester.*

Healthy volunteers (5) and patients with metastatic malignancy (9) from the following primaries (malignant melanoma 5, hypernephroma 3, and colon carcinoma) were studied. No patient had received previous systemic treatment prior to this investigation. The BCG (Glaxo) was given by Heaf gun, 5 applications per limb and blood samples taken before, and on Days 2, 4, 7, 10, 21, 28 after vaccination. The assay system employed the reaction of $^{51}$Cr-labelled Chang cells with subjects' lymphocytes alone—direct cellular cytotoxicity (DCC); with lymphocytes and rabbit anti-Chang serum—antibody-dependent cellular cytotoxicity (ADCC); and with lymphocytes stimulated by PHA—PHA-induced cytotoxicity (PC) (Holm and Perlmann, 1967, *J. exp. Med.*, **125**, 72; MacLennan and Loewi, 1968, *Nature, Lond.*, **219**, 1069. A consistent pattern of cytotoxicity was found in both volunteers and patients. At Day 2 a decrease in ADCC and PC (and also DCC in patients) was observed followed by an " overshoot " above Day 0 values in DCC, ADCC and PCC at Day 7 and 10 which declined to pre-BCG levels over the next 2–3 weeks. Those patients who partially responded to BCG therapy tended to behave like the normal volunteers, with greater overshoots and smaller " negative " phases, compared with those patients with progressive disease. The assay would appear to test both T lymphocytes (PC) and non-T lymphocyte function (ADCC) (Hersey, Edwards and Edwards, 1976, *Clin. exp. Immunol.*, **23**, 104) and describes changes in lymphocytotoxicity following immunotherapeutic endeavours.

**INHIBITION OF E-ROSETTE FORMATION BY SERA FROM CANCER PATIENTS.** R. H. WHITEHEAD, G. P. ROBERTS, J. THATCHER, C. TEASDALE and L. E. HUGHES, *University Department of Surgery, Welsh National School of Medicine, Cardiff.*

It has been shown that the percentage of E-rosetting cells is decreased in patients with carcinoma of the breast. Treatment of lymphocytes from cancer patients by mild enzyme (papain) digestion increases E-rosetting counts to normal levels. Re-incubation of these treated lymphocytes in autologous serum abrogates this effect and restores E-rosetting cell levels to pre-papain values. Incubation of lymphocytes from normal controls in sera from cancer patients also causes depression of the E-rosetting cell levels and this depression can be reversed by papain treatment. Incubation in normal allogeneic sera has less effect on E-rosetting cell levels. Fractionation studies of inhibitory sera on G200 have shown that the inhibitory factor is of high molecular weight. These findings would suggest that there is a tumour product present in the serum of cancer patients which nonspecifically masks the E-receptor sites on a proportion of T-lymphocytes. This (and other evidence) suggests that there are at least two sub-populations of human T-lymphocytes which is masked by this factor.

**LEVAMISOLE—A DOUBLE BLIND IMMUNOLOGICAL STUDY.** D. J. T. WEBSTER and L. E. HUGHES, *University Department of Surgery, Welsh National School of Medicine.*

Levamisole has been widely reported as having immunopotentiating activity in both man and animals. This paper presents the preliminary findings of an ongoing double-blind assessment of levamisole. Patients with advanced carcinoma of breast, colon, stomach or melanoma have been assessed by immunological parameters. The tests used are DNCB and Mantoux responses, total white cells and lymphocyte counts, lymphocyte stimulation by PHA and measurement of immunoglobulin classes G, A and M. Thirty-six patients have been analysed—17 levamisole and 19 placebo. No significant differences have been seen in any of the measured parameters when measured 1, 2, 3 and 6 months after a course of treatment (levamisole 3 × 150 mg/week for 4 weeks). The most frequent side-effect was giddiness, which was recorded in 5/17 patients on levamisole. There is no support in this trial for an immunopotentiating effect of levamisole in cancer patients, although larger numbers of patients require study to reach statistical significance.

**A STUDY OF THE PHARMACOKINETICS OF P-HYDROXYANILINE MUSTARD, CHLORAMBUCIL, PHENOL AND PHENYL PHOSPHATE BY HIGH-PRESSURE LIQUID CHROMATOGRAPHY (HPLC).** P. WORKMAN, J. A. DOUBLE and C. R. BALL, *Department of Cancer Research, University of Leeds.*

Simple rapid analytical methods for the determination of p-hydroxyaniline mustard (AMOH), chlorambucil, phenol and phenyl phosphate in biological materials by HPLC have been established. Only very low plasma concentrations are detectable following a supra-lethal dose of AMOH in mice, whereas chlorambucil disappears less rapidly (plasma half-life 70 min). The pharmacokinetics of phenol and phenyl phosphate were studied as model compounds for AMOH and p-hydroxyaniline mustard phosphate (AMO-phos) respectively. This work has shown (1) that phenol has a short plasma half-life (10 min) and (2) that phenyl phosphate is rapidly hydrolysed to phenol, which is detectable in high levels in plasma and tissues. This suggests that the activity of non-tumour phosphatases may negate the potential selective effect of AMO-phos on tumours possessing high phosphatase activity.

**THE CLINICAL PHARMACOLOGY OF CB 10–252 IN HEPATOMA PATIENTS.** M. H. N. TATTERSALL and G. A. CURT, *Department of Medical Oncology, Charing Cross Hospital, London.*

CB 10–252 is a latent, tissue-activated alkylating agent developed for treatment of hepatocellular carcinoma (Bukhari *et al.*, 1973, *J. natn Cancer Inst.*, **50**, 243). Hepatoma and normal liver cells contain azoreductase activity, which converts CB 10–252 into an active alkylating radical (Autrup and Warwick, 1973, *11th Int. Cancer Cong.*, **2**, 137). The active alkylating radical hydrolyses with a $T\frac{1}{2}$ of 45 s. This approximates the hepatic circulation, and CB 10–252 activated in the liver should be hydrolysed before reaching the systemic circulation and myelosuppression should not occur. An *in vitro* assay was developed to measure azoreductase activity in human tissue specimens. Human liver azoreductase activity was $4222 \pm 5\%$ nM drug reduced/g protein/15 min, bone marrow was $2437 \pm 5\%$, human spleen $2513 \pm 5\%$, and cultured human liver cells in log phase growth $2641 \pm 5\%$. Serial urine collected from 3 patients on 30 mg CB 10–252 daily showed that 80% of the drug was activated and excreted in 8 h. Trace amounts (2–5 nM/ml) of inactivated drug were identified in the plasma at this time. Rat liver, with nearly twice human azoreductase activity ($8213 \pm 5\%$), clears plasma of a 10 mg/kg i.p. injection within 2 h. Thus human hepatic enzyme activity restricts a first pass phenomenon and marrow activation of circulating drug explains myelosuppression observed in patients receiving the drug.

**HUMAN PHARMACOLOGY OF METHOTREXATE.** A. H. CALVERT and K. R. HARRAP, *Department of Applied Biochemistry, Institute of Cancer Research, Sutton.*

Methotrexate (MTX) is a widely used anticancer drug, whose toxicity, both to normal and tumour cells, is proportional to the time of exposure to the drug (Goldie, Price and Harrap, 1972, *Eur. J. Cancer*, **8**, 409). Hence the pharmacokinetics in man is an important determinant of both toxicity and therapeutic effect. Assays by the method of Bertino and Fischer (*Methods in Medical Research*, 1964; **10**, 297) reveal a biphasic plasma decay curve with half lives of approximately 0·5 and 6 h following i.v. or i.m., dosage. Renal clearance correlates well with glomerular filtration rate (GFR) and is $0{\cdot}6 \times$ GFR. Plasma clearance is about $2 \times$ GFR. The parameters for a two-compartment model have been derived from these data. A method to determine the degree of inhibition of dihydrofolate reductase (DHFR) in tissues has been developed, in an attempt to see whether $> 95\%$ inhibition corresponds with clinical response. This result would be expected from previous work (Jackson and Harrap, 1973, *Arch. Biochem. Biophys.*, **158**, 2; Harrap and Jackson, 1975, *Advances in Enzyme Regulation*, **15**, 77). Provisional data from this method indicate that a plasma MTX level of $10^{-7}$M achieves only 30% saturation of DHFR in breast carcinoma tissue.

**THE PROTECTION OF NORMAL TISSUES FROM METHOTREXATE TOXICITY.** D. C. TALBOT, J. A. STRAW, G. A. TAYLOR and K. R. HARRAP, *Department of Applied Biochemistry, Institute of Cancer Research, Sutton.*

The requirements for protection against methotrexate (MTX) toxicity with preformed purines and pyrimidines have been studied in several mammalian tumour cell lines (Tattersall *et al.*, 1974, *Eur. J. Cancer*, **10**, 819). The aim of the present study was to determine the rescue characteristics of normal proliferating tissues in order to optimize MTX treatment protocols. Male DBA2/C57BLF1 hybrid mice given lethal doses of MTX (400 mg/kg i.p.) survived when treated with thymidine, hypoxanthine and allopurinol (500, 50, 10 mg/kg i.p., thrice daily for 5 days), but were not rescued either with mixtures of hypoxanthine + allopurinol, or with thymidine alone. Mice bearing L1210 leukaemia were rescued by thymidine, indicating the ability of normal tissues to utilize low levels of purines released from autolysing tumour cells. MTX treatment induced an early purine deficiency in gut (but not marrow), though by 24 h deficiency of purine and pyrimidine was established in both tissues. These observations are of significance in relation to the selective protection of normal tissues.

**THE TOXICITY OF ADENOSINE TO LYMPHOID CELLS AND ITS POTENTIATION.** K. R. HARRAP, R. M. PAINE and J. F. SMYTH, *Department of Applied Biochemistry, Institute of Cancer Research, Sutton.*

The identification of a severe combined immunodeficiency syndrome associated with adenosine deaminase (ADA) deficiency (Dissing and Knudsen, 1972, *Lancet* ii, 1316) has focussed attention on the toxicity of adenosine to lymphocytes and the possible protective role of ADA. The particularly high level of ADA in human malignant lymphocytes may be therapeutically exploitable (Smyth, and Harrap, 1975, *Br. J. Cancer*, **31**, 544). The molecular basis of adenosine toxicity has been explored in both transplantable lymphoid tumour cells and in cultured human lymphocytes stimulated with PHA. In the former case adenosine induces imbalanced synthesis of nucleic acid and protein, while in the latter the onset of DNA synthesis is delayed. In both cases the effects of adenosine are markedly potentiated by coformycin, a tight-binding inhibitor of ADA. It is possible that combinations of adenosine + coformycin may have use, both in the treatment of acute lymphoblastic leukaemia, and in immunosuppression.

**POSSIBLE MECHANISM UNDERLYING THE SELECTIVE ANTITUMOUR EFFECT OF LEO 1031.** R. WILKINSON, E. D. GILBY, I. KONYVES and K. R. HARRAP, *Department of Applied Biochemistry, Institute of Cancer Research, Sutton, and the Research Laboratories, AB Leo, Hälsingborg, Sweden.*

Leo 1031, a prednisolone-21 ester of chlorambucil, has been shown to be less myelotoxic than chlorambucil in both animal experiments and in clinical trials. It also has increased antitumour activity, giving a 70% kill of alkylating-agent-resistant Yoshida sarcoma cells, and is more effective than chlorambucil against the sensitive Yoshida line. *In vitro*, Leo 1031 binds to a serum protein and is released as prednisolone and chlorambucil after hydrolysis by a serum esterase enzyme. Human bone marrow cells, human leukaemic blast cells and transplantable tumour cells also hydrolyse Leo 1031, but at different rates. The rate of hydrolysis in tumour cells is generally faster than in normal bone marrow or serum. This could explain the low myelotoxicity compared with chlorambucil and underlie the selective antitumour effect of Leo 1031 *in vivo*.

**QUANTIFICATION *IN SITU* OF TUMOUR RESPONSE TO CHEMOTHERAPY.** P. J. HOUGHTON and D. M. TAYLOR, *Department of Radiopharmacology, Division of Biophysics, Institute of Cancer Research, Sutton.*

A simple rapid method for the quantification of tumour response to chemotherapy *in situ* has been developed using the Lewis lung tumour and B16 melanoma in mice. This assay, based on changes in fractionation incorporation (FI) of $^3$H-thymidine into DNA, permits the calculation of an " Equivalent Repopulating Fraction " (ERF) from observations at a single time point after drug treatment. In these two tumours the ERF values agree well with the surviving fractions determined by clonogenic cell assays *in vitro*. This method appears to have general applicability to any tumour system for which the tumour doubling time can be measured and it is currently being tested in human tumour xenografts. In one such tumour, a moderately differentiated rectal adenocarcinoma, cyclophosphamide (200 mg/kg) produced a marked, but transient, depression of FI and the calculated ERF was 0·5 suggesting that the tumour was relatively resistant to this agent.

**RESPONSE OF THE EMT6 MOUSE TUMOUR TO CYCLOPHOSPHAMIDE —A COMPARISON OF RESULTS OBTAINED USING GROWTH CURVE DATA AND THE *IN VITRO* PLATING TECHNIQUE.** P. R. TWENTYMAN, *MRC Clinical Oncology and Radiotherapeutics Unit, The Medical School, Hills Road, Cambridge.*

Following treatment of EMT6 solid tumours with cyclophosphamide at a volume of either 50 or 300 mm$^3$, a growth delay of around 3 days per 100 mg/kg (in the range 0–300 mg/kg) is produced. Results of an experiment measuring time to reach a given tumour volume following various inocula indicate a minimum tumour doubling time of 20–22 h. The greatest reduction in surviving fraction compatible with a growth delay of 3 days is therefore around a factor of 10x for each 100 mg/kg. Experiments in which cell suspensions are prepared from treated tumours and plated out *in vitro* indicate surviving fractions which are lower than this. Examination of changes in measured surviving fraction over the first 48 h after treatment strongly suggest that the operation of " recovery from potentially lethal damage " may explain these discrepancies. Experiments on tumours treated whilst still at the undetectable stage indicate that the growth delay for a given dose of cyclophosphamide is increased, and therefore that the reduction in surviving fraction is probably more severe in these sub-clinical tumours.

**COLLATERAL SENSITIVITY STUDIES BETWEEN HALOGENATED METHOTREXATES AND AN ALKYLATING AGENT.** B. W. FOX and D. J. PILLINGER, *Paterson Laboratories, Christie Hospital and Holt Radium Institute, Manchester.*

A few examples of collateral sensitivity have now been described in mammalian tumour cell lines. The phenomenon, originally observed in bacterial systems, describes the simultaneous sensitization of a tissue to one drug on acquiring resistance to another. Most reported examples have been demonstrated in whole animals, and may thus be influenced by changes in the host. Furthermore, the few recorded instances have been associated primarily with resistance towards antimetabolites. In the work to be presented, an example of collateral sensitivity towards halogenated methotrexates of tumours resistant to an alkylating agent, methylene dimethane sulphonate will be described. In this system, collateral sensitivity, observed in whole animal studies, is maintained when the tumours are grown in culture as continuous cell lines. The level of dihydrofolate reductase has been measured in these cell lines and the possible origin of the collateral sensitivity will be discussed.

**THE EFFECT OF CYCLOPHOSPHAMIDE AND METHOTREXATE ON " SALVAGE " AND *DE NOVO* DNA SYNTHESIS.** K. D. TEW and D. M. TAYLOR, *Radiopharmacology Dept., Institute of Cancer Research, Sutton.*

After single-dose methotrexate (MTX) or cyclophosphamide (CY) treatment the uptake of $^3$H-thymidine (salvage), $^3$H-deoxyuridine (*de novo* thymidine) and $^{14}$C-formate (*de novo* thymine and purines) has been monitored in the BICR A15 rat tumour as compared to gut and bone marrow. Treatment with 100 mg/kg CY has shown a potentially exploitable time differential between recovery of vital tissues (3–5 days) and tumour ($>$ 10 days).

Methotrexate has been shown to exert its cytotoxic effect by depleting *de novo* thymine production rather than *de novo* purine synthesis. This can be linked biochemically with the ability of the latter pathway to recycle tetrahydrofolic acid and overcome the MTX block. Tumour cells efficient in salvaging thymidine, can overcome the "*de novo*" thymine block more effectively than gut cells—a fact which may limit the therapeutic usefulness of MTX.

### OBSERVATIONS ON THE PREVENTION OF CYCLOPHOSPHAMIDE-INDUCED CYSTITIS.

D. A. Tolley and J. E. Castro, *Urological Unit, Royal Postgraduate Medical School, Hammersmith Hospital, London.*

The clinical use of cyclophosphamide may be severely limited by its toxic effects on the urinary bladder (Spechter *et al.*, 1965, *Dtsch. med. Wschr.*, **20**, 1458). We have developed a laboratory model in the rat in which these toxic phenomena are consistently reproduced. The effects of N-acetylcysteine on the prevention and treatment of cyclophosphamide-induced cystitis were studied, and our observations showed that N-acetylcysteine was effective in the prophylaxis and early treatment of this condition. The results of cyclophosphamide and N-acetylcysteine therapy on peripheral white count, spleen morphology and weight showed that the effect of combined therapy and cyclophosphamide alone were identical. Such observations suggest that N-acetylcysteine does not interfere with the cytotoxic effects of cyclophosphamide.

### MECHANISTIC STUDIES WITH A CELL LINE RESISTANT TO ICRF 159.

K. White and A. M. Creighton, *Imperial Cancer Research Fund, Lincoln's Inn Fields, London.*

A line of BHK21S cells has been isolated which shows resistance to the anti-tumour agent ICRF 159, and has been designated BS/159–1. The resistance index of BS/159–1 cells is 40 as determined by *in vitro* survival assays. Resistant cells have similar growth characteristics to the sensitive parent line. The resistance is not due to decreased uptake or altered metabolism of the drug. In contrast to the sensitive line, BS/159–1 cells enter mitosis at the normal rate, and have discretely condensed chromosomes when treated with doses of ICRF 159 up to 100 μg/ml. At higher doses, characteristic toxic effects are seen. Studies with a wide variety of cytotoxic agents (using *in vitro* survival assays) show the same degree of cross resistance only to structural analogues of ICRF 159. A smaller but consistent cross resistance to adriamycin and daunomycin was demonstrated.

### CHEMOTHERAPY AS PRIMARY TREATMENT FOR SMALL CELL CARCINOMA OF THE LUNG.

P. K. Bondy and E. D. Gilby, *Biology of Human Cancer Unit, Ludwig Institute for Cancer Research, Royal Marsden Hospital, London.*

Sixty-one patients with small-cell carcinoma were treated with a 5-drug chemotherapy schedule given every 4 weeks. Tumour regression to at least 50% of pre-treatment size occurred in 60% of cases. All patients also received radiotherapy. The survival of chemotherapy responders was significantly greater (median 44 weeks) than that of non-responders (median 19 weeks). No difference in median survival was seen between patients treated initially with radiotherapy and those in whom it was deferred until after the second course of chemotherapy, but it was frequently impossible to assess responsiveness to drugs after radiotherapy. Since the median survival of all patients studied was not significantly greater than that of a group treated by deep X-ray alone we conclude that this disease should initially be treated with chemotherapy alone to select those in whom further chemotherapy is worthwhile. Also the role of radiotherapy in these patients needs further study.

### IMPAIRED WATER EXCRETION IN OAT-CELL LUNG CANCER.

E. D. Gilby, P. K. Bondy and M. Forsling, *Biology of Human Cancer Unit, Ludwig Institute of Cancer Research, Royal Marsden Hospital, London and Department of Physiology, Middlesex Hospital Medical School, London.*

Water load tests and plasma arginine vasopressin (AVP) assays were performed on 49 patients with untreated oat-cell carcinoma of the lung. Only 15 patients displayed a normal diuretic response with normal suppression of plasma AVP. The syndrome of inappropriate antidiuretic hormone secretion was common, and usually asymptomatic. Its presence in 17 patients (35%) was confirmed by observing elevated plasma AVP with hypotonic plasma and concentrated urine. The syndrome resolved after successful anti-tumour chemotherapy but persisted in patients who showed no tumour regression. In the remaining 17 patients lesser degrees of impaired water excretion occurred, with variable AVP levels, possibly resulting from stress of the illness. Plasma AVP levels do not provide a reliable tumour marker in this disease, but the syndrome of inappropriate secretion is worth seeking, since it is common, may be a guide to success of treatment and may require treatment *per se*.